# Baseline PARP-1 PET imaging in patients with advanced solid tumors with DNA damage response mutations

**DOI:** 10.1007/s00259-025-07595-3

**Published:** 2025-11-18

**Authors:** Tarek Daoud, Jiansong Chen, Peng Wei, Franklin Wong, Timothy A. Yap, Lilie L. Lin

**Affiliations:** 1 Department of Radiation Oncology, The University of Texas MD Anderson Cancer Center, Unit 1422, 1515 Holcombe Blvd, Houston, Houston, TX 77030 USA; 2 Department of Biostatistics, The University of Texas MD Anderson Cancer Center, Houston, TX USA; 3 Department of Nuclear Medicine, The University of Texas MD Anderson Cancer Center, Houston, TX USA; 4 Department of Investigational Cancer Therapeutics (Phase I Program, The University of Texas MD Anderson Cancer Center, Houston, TX USA; 5 Therapeutics Discovery Division, The University of Texas MD Anderson Cancer Center, Houston, TX USA

**Keywords:** [^18^F]FluorThanatrace, FTT, PARP inhibitors, BRCA, PET imaging biomarker

## Abstract

**Purpose:**

Inhibitors of poly(ADP-ribose) polymerase (PARP), an enzyme with numerous roles in DNA damage response signaling, represent a class of anti-cancer drugs approved for treating solid tumors with defects in the DNA damage response. However, additional methods for identifying patients who would benefit from the use of PARP inhibitors are urgently needed. We evaluated a novel radiotracer, ^18^F-FluorThanatrace ([^18^F]-FTT), to noninvasively assess PARP activity with PET/CT before treatment and determine associations between uptake, tumor mutation status, and prior receipt of therapy including PARP inhibitors.

**Methods:**

Fifty-two patients with solid tumors underwent whole-body (skull base to thigh) [^18^F]-FTT PET/CT scans before beginning PARP inhibitor treatment. The maximum standardized uptake value (SUVmax) was recorded for up to five lesions per patient. All recorded lesions were included in the analysis.

**Results:**

Uptake of [^18^F]-FTT was confirmed in all 52 patients regardless of primary tumor type (20 breast, 12 ovarian, 7 prostate, 3 pancreas, and 10 other). Lesion-by-lesion analysis revealed significant differences in [^18^F]-FTT uptake by primary tumor type (*P* < 0.001) and higher uptake in lesions with *BRCA2* mutations (*n* = 65) than in lesions with other genetic mutations (*n* = 26) (6.7 vs. 5.5, *P* = 0.03). Prior receipt of PARP inhibitor was associated with lower SUVmax (4.7 vs. 6.4, *P* = 0.001), and prior receipt of systemic therapy was also associated with lower SUVmax (4.0 vs. 6.0, *P* = 0.01).

**Conclusions:**

[^18^F]-FTT PET/CT may be useful as a noninvasive quantitative assessment of PARP1 enzyme activity in patients with solid tumors; uptake of [^18^F]-FTT varies based on tumor mutational status and receipt of prior PARP inhihbitor therapy and/or systemic therapy.

## Introduction

One of the major pathways for DNA repair is homologous recombination (HR), a high-fidelity multistep process in which DNA double-stranded breaks occurring during DNA replication are detected and repaired [1]. Defects in the HR repair pathway have been described in several tumor types, with the highest prevalence in breast, ovarian, prostate, and pancreatic cancers [2]. In a meta-analysis that included 6 studies of 21,842 patients with pancreatic cancer, the prevalence of HR defects ranged from 14.5% to 16.5% when assessed by targeted next-generation sequencing (NGS) and 24% to 44% by whole-exome sequencing [3]. Another group used NGS to analyze 17,566 tumors and found the prevalence of HR defects to be 31.9% to 36.9% in endometrial cancer, 18.5% to 21.6% in ovarian cancer, 13.9% to 17.5% in breast cancer, and 15.2% to 21.2% in melanoma [4].

Among the numerous genes involved in HR repair are the tumor suppressors *BRCA1* and *BRCA2*, which are mutated in 2% to 4.7% of all solid tumors [4, 5]. A key enzyme in the DNA repair process in humans is poly(ADP-ribose) polymerase (PARP), which detects DNA strand breaks, regulates the choice of DNA repair pathways, and influences the efficiency of DNA repair [6]. Inhibiting PARP-1 in tumors with *BRCA1 or BRCA2* mutations leads to tumor cell death through synthetic lethality because the cells are unable to use HR to repair damaged DNA [6, 7]. In pivotal preclinical studies, cells deficient in *BRCA1* or *BRCA2* were found to be up to 1000 times more sensitive to PARP inhibitors [1, 8] a discovery that led to the clinical testing of PARP inhibitors and their subsequent approval by the US Food and Drug Administration for selected patients with breast, ovarian, pancreatic, and prostate cancer.

Beyond *BRCA1* or *BRCA2* mutations, identifying those patients who would gain the most benefit from PARP inhibitors remains a challenge. Furthermore, not all tumors with defects in *BRCA1/2* or HR repair respond to PARP inhibitors, and certain tumors with intact *BRCA1/2* genes do respond to PARP inhibitors [9]. In vitro data have shown that PARP-1 levels correlate positively with cytotoxicity from PARP inhibitors [10], whereas loss of PARP-1 significantly reduces PARP inhibitor cytotoxicity [11]. However, measuring PARP-1 clinically is complex because current methods, such as immunohistochemical staining, require tumor tissue sampling from patients, which in turn limits the assessment to a single time point and provides little information on tumor heterogeneity. Thus a noninvasive, quantitative means of measuring PARP-1 activity in tumors serially during therapy as a putative biomarker of sensitivity to PARP inhibitors is urgently needed.

One minimally invasive method of measuring PARP-1 activity in tumors over time is through the use of novel radiotracers with whole body imaging with positron emission tomography (PET), such as [^18^F] FluorThantrace ([^18^F]-FTT), a recently developed highly specific novel PET tracer that measures PARP-1 expression [12]. We previously tested [^18^F]FTT-PET/CT for patients with suspected or known epithelial ovarian cancer and found that high [^18^F]-FTT uptake was localized to areas identified as disease on radiographic imaging (CT with contrast and/or [^18^F]-FDG PET/CT). Moreover, the standardized uptake values (SUV) of [^18^F]-FTT were found to correlate positively with PARP-1 levels in tumor tissue, as measured by fluorescent immunohistochemical staining and autoradiography [13]. Other studies of patients with breast cancer demonstrated that [^18^F]-FTT can be effectively and safely used as a noninvasive biomarker of PARP expression [14]^,^ [15]. Collectively, these early studies highlight the potential of [^18^F]-FTT for use as a minimally invasive biomarker of PARP-1 expression and support the further investigation of [^18^F]-FTT PET/CT as a predictive biomarker of response to treatment [13]. As the studies to date have mainly focused on patients with breast, prostate, or ovarian cancers, we sought to determine whether uptake could be observed in patients with other solid tumors and whether levels of uptake of [^18^F]-FTT in tumor could be associated with other patient or treatment related variables.

This prospective, single-institution study aimed to assess the variability of uptake of [^18^F]-FTT in patients with primary or metastatic solid tumors and DNA damage response (DDR) mutations who were beginning primary or next-line therapy for their solid tumor. Such an assay would potentially provide clinicians with a noninvasive imaging tool to guide patient selection for PARP inhibitor–based therapeutic regimens.

## Methods

This prospective study was conducted at The University of Texas MD Anderson Cancer Center after receiving institutional review board approval (clinical trial identifier: NCT03604315) from the “IRB and Scientific Review Board Committee” at MD Anderson Cancer Center. All participants provided written informed consent to participate in this study. Our research was conducted in accordance with the ethical principles outlined in the National Commission for the Protection of Human Subjects of Biomedical and ehavioral Research known as “The Belmont Report.” Eligible patients were 18 years or older with solid tumors and at least one lesion measuring 1 cm on standard imaging (CT, MRI, ultrasound, or FDG PET/CT) as per RECIST 1.1 [16]. Patients were scanned prior to any oncologic therapy if newly diagnosed or with newly recurrent disease; for patients who were switching systemic therapy regimens due to progression, they were imaged prior to initiating their new regimen and at least 2 weeks since any systemic therapy. Patients were excluded if they could not tolerate lying flat for >45 min. Patients were recruited and enrolled from February 2019 to January 2023.

Patients underwent whole-body static [^18^F]-FTT PET/CT scans from the skull base to the thigh. A single intravenous dose of [^18^F]-FTT (median dose 10.04 mCi/371.48 MBq [range 7.4–11.34 mCi/273.8–419.58 MBq]) was administered, and PET/CT scans were obtained 90 min after injection by using a GE Discovery 690 FX whole-body PET/CT scanner; the timing of imaging was based on our prior studies which have demonstrated correlation of uptake on PET at 90 min post injection and PARP-1 imunofluorescence [13]. Vital signs including heart rate, systolic and diastolic blood pressure, and temperature were assessed before [^18^F]-FTT injection and after PET/CT scanning. Patients were contacted by telephone within 24 h (or on the next business day) after the injection to monitor any adverse events and for adverse event attribution.

Initial imaging from the skull base to mid-thigh was used to assess regional [^18^F]-FTT uptake in tumor tissue. Acquired PET/CT images were reviewed by using MIM version 7 (MIM Software Inc., Cleveland, OH). Imaging studies were reviewed by one radiologist (TD) and confirmed by a second nuclear medicine physician (FW). All areas of abnormal non-physiologic uptake > 1 cm were identified and reviewed in conjunction with other available cross sectional imaging to confirm pathologic lesions (i.e. CT, MR, [^18^F]Fluorodeoxyglucose-PET imaging). Up to 5 lesions were identified and recorded for each patient with a maximum of two metastases chosen within the same organ; cystic lesions were avoided. Maximum standardized uptake value (SUVmax) normalized to body weight was recorded for all lesions.

All patients’ electronic medical records were reviewed through EPIC (cloud-based electronic medical records software) and entered into a secure, password-protected REDCap database. Clinicopathologic, treatment, laboratory, baseline tumor markers and imaging data were collected from the medical records. Somatic and germline mutation testing results were reviewed for all patients, and mutations in *BRCA1* and *BRCA2*, as well as other DDR mutations including *ARID1A*,* ATM*,* ATR*,* FANCC*,* CDK12*,* PALB2*, and *RAD51* were recorded. All genetic results were obtained from Clinical Laboratory Improvement Amendments (CLIA)-certified labs as part of routine clinical care.

### Statistical analysis

We conducted a lesion-by-lesion analysis that considered lesions independently (i.e., not by patient), with Wilcoxon rank-sum tests used to evaluate correlations between [^18^F]-FTT uptake (I.e., SUVmax) and mutation status of DDR genes, prior systemic regimens, and prior PARP inhibitor treatment. Kruskal-Wallis tests were also used to assess the relationship between [^18^F]-FTT uptake and site of primary disease (i.e. breast, prostate, ovary, etc.) and lesion site (i.e. node, lung, liver, bone, etc.). Interlesional heterogeneity was calculated using the standard deviation of SUVmax within each patient.

## Results

### Patient characteristics

Fifty-two patients with solid tumors (median age 56; range 27–81) were enrolled from February 2019 to January 2023. The most common cancer types were breast (*n* = 20), ovarian (*n* = 12), prostate (*n* = 7), and pancreatic cancer (*n* = 3) (Table 1). Fifteen patients (29%) had received a prior PARP inhibitor and 31 patients (60%) had previously received at least one systemic treatment regimen. No patients were on a PARP inhibitor regimen at the time of recruitment. Among the 52 patients, 44 had *BRCA1/2* mutations (23 somatic, 33 germline), 24 had *TP53* mutations, and 32 had mutations in other genes (e.g., *KRAS*,* CHEK2*,* RAD51*).

**Table 1:** Baseline patient demographics and tumor characteristics

Characteristics	Value or No. of Patients (%)
All Patients	52 (100)
Age at Time of Study, years	
Mean (SD)	56 (± 13.2)
Range	27.3–81
Sex	
Male	13 (25)
Female	39 (75)
Race	
White	41 (79)
Black	2 (4)
Asian	1 (2)
Other	8 (15)
Primary Cancer Site	
Breast	20 (38)
Ovary/Primary Peritoneal	12 (23)
Prostate	7 (13)
Pancreas	3 (6)
Endometrium	3 (6)
Peritoneum	2 (4)
Leiomyosarcoma	1(2)
Vaginal melanoma	1 (2)
Urinary bladder	1 (2)
Kidney	1 (2)
Rectum	1 (2)
No of Prior Systemic Regimens for Each Patient	
0	21 (40)
>1	31 (60)
Previous PARP Inhibitor Treatment	
Yes	15 (29)
No	37 (71)
Pathogenic DDR Pathway Mutations	
BRCA1	23 (44)
BRCA2	21 (40)
Other DDR pathway mutations	8 (15)
Lesion location	
Nodal	52 (29.5)
Liver	27 (15.3)
Lung	5 (2.8)
Bone	18 (10.2)
Breast	7 (4.0)
Ovary	10 (5.7)
Other	57 (32.5)

*DDR* DNA damage response

### [^18^F]-FTT uptake by primary disease site

**Fig. 1:**
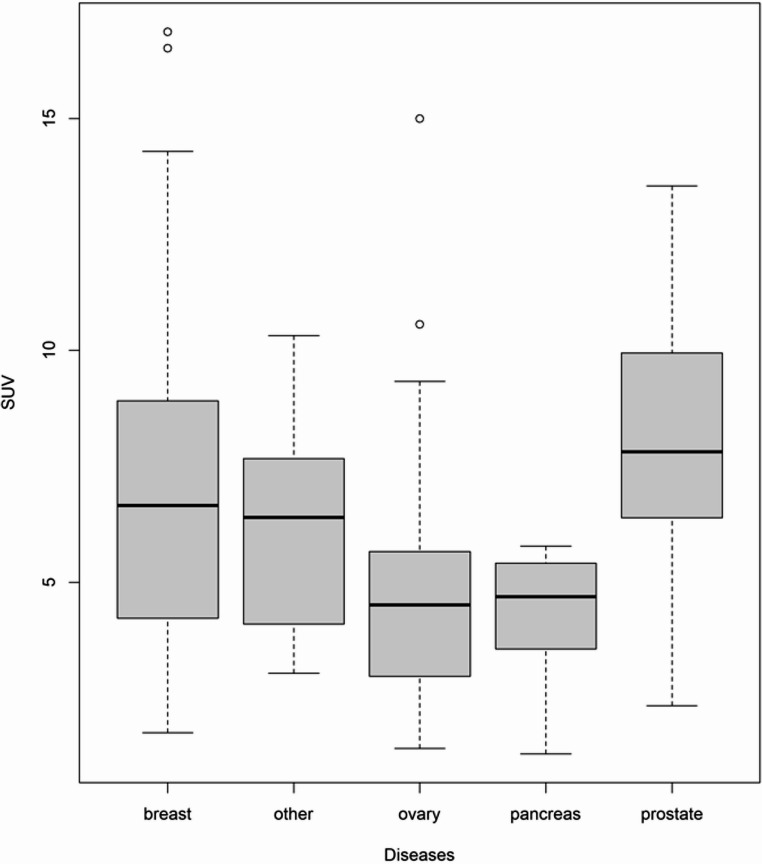
Boxplot of SUVmax in all lesions by primary cancer site

**Fig. 2:**
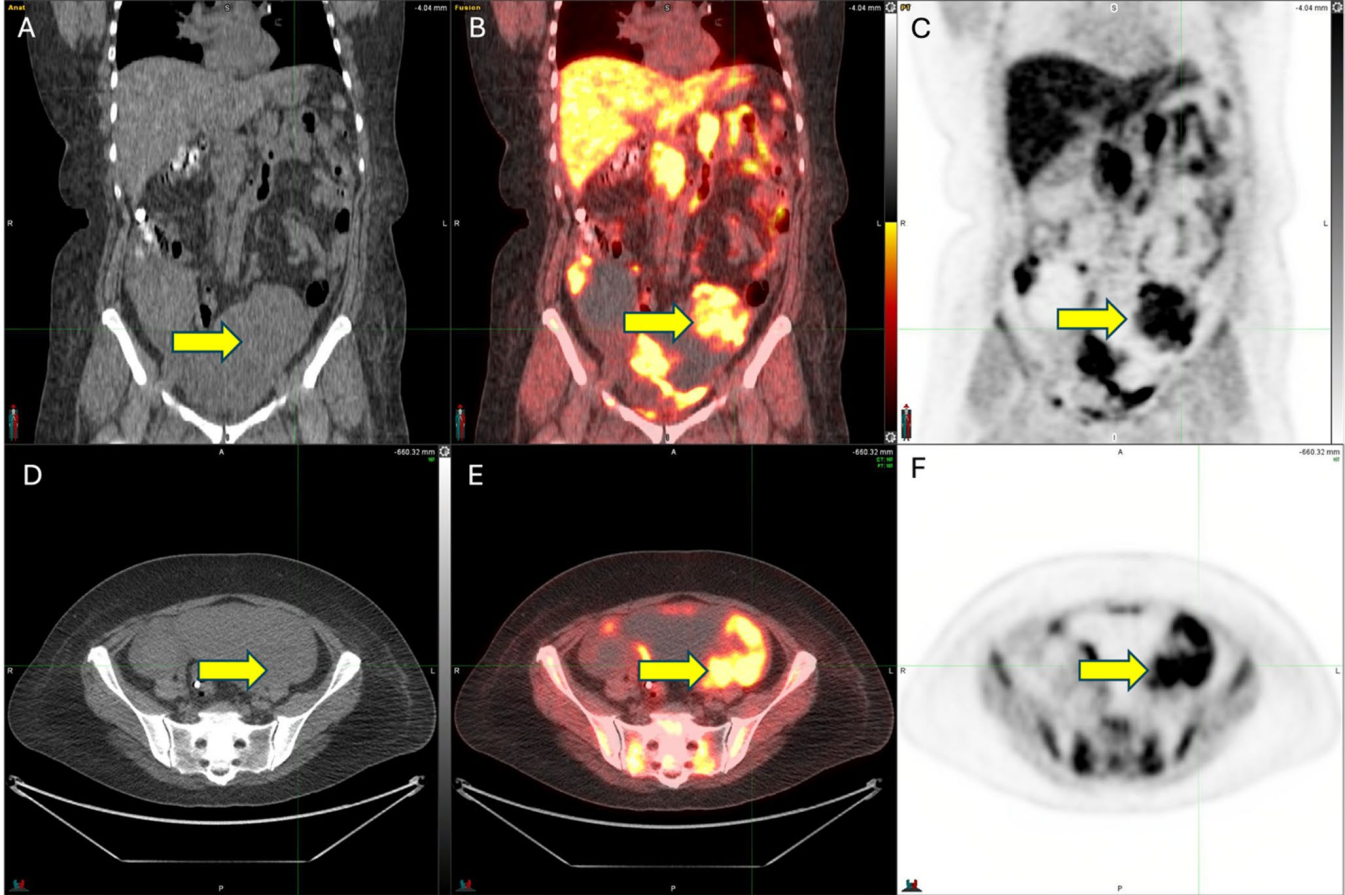
Images are from a 52-year-old woman with a germline *BRCA1* mutation high-grade serous ovarian cancer showing avid ^18^F-FTT uptake in the left ovary (outlined in purple, yellow arrow) on coronal Non-Contrast CT (**A**), [^18^F]-FTT PET/CT (**B**) and [^18^F]-FTT PET (**C**) and axial Non-Contrast CT (**D**), [^18^F]-FTT PET/CT (**E**) and [^18^F]-FTT PET (F) with SUVmax = 9 (green circle refers to point of maximum activity), tumor to muscle ratio (T/M) = 4.9

Measurable [^18^F]-FTT uptake was observed in all patients regardless of type of primary disease (i.e. breast, ovary, prostate, etc.). Our lesion-by-lesion analysis (176 lesions total) revealed significant differences in median SUV among the different types of primary tumors (*P* < 0.001; Table 2; Fig. 1). In pairwise comparisons, median SUVmax in primary breast lesions (6.66 [range 1.76–16.9]) significantly differed from median SUVmax in primary ovarian lesions (4.51 [range 1.42–15], *P* = 0.001) and lesions of patients with primary pancreatic tumors (4.51 [range 1.42–15], *P* = 0.048). However, median SUVmax did not differ between primary breast lesions and lesions of patients with primary prostate tumors (median 7.82 range [range 2.34–13.6], *P* = 0.14). Distribution of lesion sites in patients are outlined in Table 1; there was a significant difference overall in uptake by lesion location (Table 2). Representative images from a patient enrolled in this study are shown in Fig. 2.

**Table 2:** SUVmax by primary tumor site and lesion location

Primary tumor site	Number of Lesions	SUVmax Median (Range)
Breast	63	6.66 (1.76–16.9)
Pancreas	7	4.69 (1.3–5.78)
Prostate	24	7.82 (2.34–13.6)
Other cancers	35	6.4 (3.04–10.3)
Lesion location	Number of Lesions	SUVmax Median (Range)
Nodal	52	6.48 (2.32–16.87)
Liver	27	6.35 (2.78–9.96)
Lung	5	3.25 (2.85–4.39)
Bone	18	8.19 (4.00–12.61)
Breast	7	3.61(1.76–5.75)
Ovary	10	7.27(3.90–9.33)
Other	57	4.45 (1.30–13.55)

P-value of Kruskal-Wallis test for SUVmax across lesion locations was less than 0.001

### [^18^F]-FTT uptake by mutations in DDR genes

Next we stratified SUVmax according to mutations in DDR genes. Patients were grouped as having mutations in *BRCA1* (*n* = 23) vs. *BRCA2* (*n* = 21) vs. other non-BRCA DDR pathway genes (*n* = 8) as outlined above. Again, measurable [^18^F]-FTT uptake was observed in all patients regardless of tumor mutation status. In lesion-by-lesion analysis, lesions of patients with mutated *BRCA2* (*n* = 65) had higher median SUVmax than lesions of patients with other mutations (*n* = 26), 6.7 vs. 5.5, *P* = 0.033. In lesion-by-lesion analysis, SUVmax did not differ in lesions of patients who had *BRCA1* (*n* = 85) vs. *BRCA2* (*n* = 65) mutations, 6.25 vs. 6.69 (*P* = 0.51), or in lesions of patients who had mutations in BRCA1 vs. other DDR genes, 6.25 vs. 5.20 (*P* = 0.18). A non-significant difference in uptake may have been present when *BRCA1*/*BRCA2* mutations (*n* = 150) vs. other non-BRCA DDR pathway gene mutations (*n* = 26) were compared (*P* = 0.069).

### [^18^F]-FTT uptake by prior receipt of therapy

**Fig. 3:**
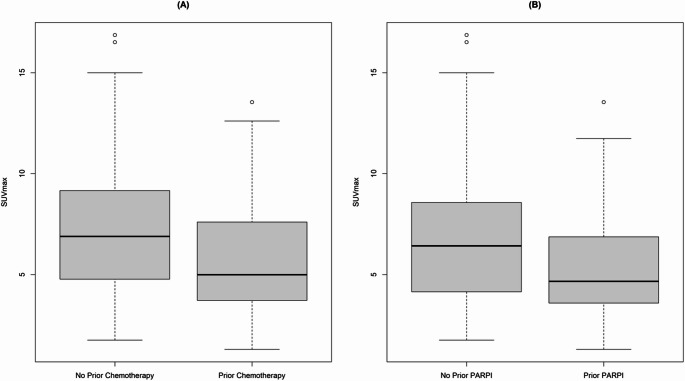
Boxplot of SUVmax for all lesions grouped by receipt of prior systemic therapy (**A**) and prior treatment with PARP inhibitors (**B**). Lesions from patients who had not received prior systemic therapies had a significantly higher SUVmax (6.9 vs 5, *P*=0.01); likewise, lesions from patients without prior PARP inhibitor treatment had a higher SUVmax (6.4 vs 4.7, *P*=0.01), relative to lesions from patients who had not received either type of therapy

We also used lesion-by-lesion analysis to evaluate [^18^F]-FTT uptake according to prior receipt of systemic therapy (*n* = 115) or no systemic therapy (*n* = 61). The median SUVmax was significantly higher in lesions from patients who had not received systemic chemotherapy (6.9 vs. 5.0, *P* = 0.01) (Fig. 3A); however, no significant difference was found by number of prior systemic regimens (data not shown). Lesions in PARP inhibitor-naïve patients (*n* = 115) showed significantly higher SUVmax than lesions in patients who had received PARP inhibitors (*n* = 61, 6.4 vs. 4.7, *P* = 0.01) (Fig. 3B). We also examined interlesional heterogeneity. The median variance of SUVmax was 2.62 (0.137-36.0). The interlesional heterogeneity did not differ by number of past regimens (0 vs. 1 vs. ≥ 2) or by receipt of prior PARP inhibitor therapy.

### Adverse events associated with [^18^F]-FTT

The most common adverse event, experienced by 9 patients (20%), was transient hypertension, followed by grade 1 nausea (2 patients [4%]), grade 1 sinus tachycardia (2 patients [4%]), and pain (grade 1 in 2 patients [4%], grade 2 in 1 patient, and grade 3 in 1 patient). One patient experienced a grade 1 allergic reaction, which developed within 24 h of her imaging and resolved within 48 h; it was determined to be possibly related to the [^18^F]FTT. Another had a grade 2 fever which was scored as unlikely related to [^18^F]FTT. None of the adverse events were considered clinically significant.

## Discussion

Our findings confirm those from previous studies of patients with breast, prostate, and ovarian cancer and expand the investigation of a novel PARP inhibitor PET agent [^18^F]-FTT to other types of solid tumors, including melanoma, leiomyosarcoma, and uterine carcinoma. In this cohort of patients enriched for DDR mutations, we observed measurable [^18^F]-FTT uptake in all patients regardless of primary disease site, mutation status, or previous receipt of a PARP inhibitor. As far as we are aware, this study represents the first instance of establishing a connection between prior administration of systemic therapies, PARP inhibitors, and the uptake of [^18^F]-FTT across a diverse range of solid tumor types. Our study builds on our two previous feasibility studies in which [^18^F]-FTT was used to image tumors in women with (1) known or suspected epithelial ovarian cancer [13] and (2) newly diagnosed breast cancer receiving neoadjuvant talazoparib [17]. In our initial ovarian tumor pilot study, [^18^F]-FTT uptake was also observed in all patients, and tumor uptake was found to correlate with ex vivo measures of PARP-1 expression in a small subset of patients (*n* = 7) who had tumor biopsy samples available for analysis.

In addition to breast and ovarian tumors, the feasibility of using [^18^F]-FTT-PET/CT to image patients with metastatic castration-resistant prostate cancer was demonstrated by Dehdashti et al. [18]. The range of [^18^F]-FTT uptake in that study was broad (SUVmax 2.3–15.4), with the highest [^18^F]-FTT uptake recorded in patients with a pathogenic *BRCA2* variant; patients with DDR defects had a significantly higher SUVmax than did patients with no defects (*P* = 0.03) [18]. In contrast to that study’s cohort of nine patients that included patients both with and without DDR defects [18], our subset of seven patients consisted exclusively of individuals with DDR defects. Our own findings from those 7 patients with prostate cancer showed a similar, although slightly lower range ([^18^F]-FTT SUVmax 3.5–12), which could be explained by the heterogeneity of tumors across the cohorts and the multiple prior regimens received by our patients with prostate cancer.

In a lesion-by-lesion analysis of the entire cohort, we also observed higher [^18^F]-FTT uptake in lesions from patients with *BRCA2* mutations (*n* = 65) than in those with mutations in other DDR pathway genes (*n* = 26, *P* = 0.03), though we did not observe differences between BRCA1m vs. BRCA2m lesions. Differential sensitivity to PARP inhibitors by DDR mutations including *BRCA1* vs. *BRCA2* has been previously described in patients with prostate cancer by Abida et al. in a prospective clinical trial testing rucaparib in patients with metastatic castrate resistant prostate cancer with higher PSA (prostate specific antigen) response rate observed in patients with *BRCA2* alterations [19], and potential explanations for this have been postulated [20]. In a multicenter retrospective analysis of 123 patients with metastatic castrate-resistant prostate cancer who had received treatment with a PARP inhibitor (i.e., rucaparib, olaparib, veliparib, or talazoparib), patients with either somatic or germline *BRCA2* mutations had longer PSA progression-free survival (PFS) (HR, 1.94; 95% CI, 0.92 to 4.09; *p* = 0.08), PFS (HR, 2.08; 95% CI, 0.99 to 4.40; *p* = 0.05), and overall survival (HR, 3.01; 95% CI, 1.32 to 6.83; *p* = 0.008) time than did patients with *BRCA1* mutations [21]. Collectively, these findings support further investigation of [^18^F]FTT in clinical trials of PARP inhibitors as a biomarker that measures PARP1 expression and sensitivity to PARP inhibitors.

In a study by Edmonds et al., in vitro and in vivo studies of breast cancer cell lines in cultured mouse embryonic fibroblast and tumor-bearing mouse models showed positive correlations between PARP expression and [^18^F]-FTT uptake, and lower [^18^F]-FTT uptake in wild-type *BRCA1/2* cell lines than in *BRCA1* mutated cell lines [22]. Our current study included patients with both somatic and germline *BRCA1/2* gene mutations as well as patients with mutations in other genes including *ARID1A*,* ATM*, and *RAD51*. Lesions from patients with *BRCA2* mutations showed significantly higher uptake than lesions from patients with mutations in other DDR genes, regardless of the primary tumor site (*P* = 0.006). Prior in vitro studies by Hopkins et al. [11] provide evidence of the relationship between levels of PARP-1 and cytotoxicity due to PARP inhibitors, supporting further testing of [^18^F]-FTT PET/CT as a method of measuring PARP-1 levels as a noninvasive way to select patients whose tumors may be sensitive to PARP inhibitors. Further, our group recently demonstrated a potential relationship between uptake of [^18^F]FTT in bone marrow and hematologic toxicity in patients with newly diagnosed BRCAm breast cancer receiving neoadjuvant talazoparib, which also warrants further investigation [17].

McDonald et al., analyzing [^18^F]-FTT uptake in 30 patients with primary breast tumors, observed a relatively broad range in SUVmax (2.6–11.3) that was independent of receptor status or the presence of *BRCA* pathogenic variants [15]. In our own study of 20 patients with breast cancer, the range of [^18^F]-FTT uptake was broader (SUVmax 1.8–16.5); we suspect that the large range reflects our inclusion of not only patients with newly diagnosed primary breast cancer but also others with metastatic disease that had been treated with several lines of prior therapy. Moreover, lesion-by-lesion analysis of tumors with *BRCA2* mutations, including those in breast and other primary sites, revealed significantly higher SUVmax than in patients with mutations in other DDR genes, a finding similar to that observed in patients with BRCA2m breast cancer in the analysis by McDonald et al. [15].

A recent study by Pantel et al. examined the use of [^18^F]-FTT PET/CT in 16 patients with high-grade serous ovarian carcinoma before treatment with a PARP inhibitor, either alone or with another agent with synergistic activity (e.g., ataxia telangiectasia mutated and Rad3-related kinase [ATR] inhibitors) [23]. The range in SUVmax range at baseline for these patients was 1.7–10.2 [23], which was similar to that in the current study of 12 patients with ovarian cancer (2.1–9.3). Both cohorts similarly had 4 patients who had received prior PARP inhibitor treatment.

Our patient cohort encompassed various cancer types, including leiomyosarcoma, melanoma, urinary bladder cancer, kidney cancer, and rectal cancer, and is the largest clinical study to date using [^18^F]FTT PET in patients with solid tumors. However, because of the limited number of patients in each subgroup (one patient per subgroup in some of the less common malignancies) and the heterogeneity of our study group (both primary and metastatic tumors), further studies with larger patient populations are necessary to validate our findings. A strength of our cohort is the availability of detailed genetic and tumor mutational information on *BRCA* genes or other non-BRCA DDR pathway gene mutations. Our study specifically examined the baseline pretreatment imaging of patients with solid tumors; examining any association between uptake on [^18^F]-FTT PET and response to treatment is beyond the scope of this analysis. To gain further insights for treatment decision-making, studies of serial [^18^F]-FTT PET for patients receiving PARP inhibitor monotherapy or combination treatment are ongoing.

We demonstrated uptake in a broad variety of solid tumors by using [^18^F]-FTT PET. To our knowledge, this study is the first to link previous receipt of systemic therapies and PARP inhibitors with [^18^F]-FTT uptake. Specifically, we noted that [^18^F]-FTT uptake was higher in lesions of patients without previous systemic therapy or PARP inhibitors. Whether these patients have a greater sensitivity to PARP inhibition or similar agents is beyond the scope of this analysis. However, serial [^18^F]-FTT PET/CT studies in patients receiving PARP inhibitors (*14*) indicate that uptake of [^18^F]-FTT could be blocked by the presence of PARP inhibitors [17, 23], suggesting that [^18^F]-FTT could be a pharmacodynamic biomarker of target engagement in addition to being a predictive biomarker for response to PARP inhibitors, alone or with other therapies. These topics are the subject of future investigations by our group, and the findings could be informative in guiding treatment decisions.

In summary, our study broadens the application of [^18^F]-FTT PET/CT imaging beyond breast, ovarian, and prostate cancers, encompassing a range of solid malignancies including those with mutations in other DDR pathway genes. Our findings also shed light on factors influencing [^18^F]-FTT uptake, such as prior systemic therapies including PARP inhibitors and mutations in DDR pathway genes.

## Data Availability

The data will be available upon reasonable request.
